# Gene Mutation in MicroRNA Target Sites of CFTR Gene: A Novel Pathogenetic Mechanism in Cystic Fibrosis?

**DOI:** 10.1371/journal.pone.0060448

**Published:** 2013-03-26

**Authors:** Felice Amato, Manuela Seia, Sonia Giordano, Ausilia Elce, Federica Zarrilli, Giuseppe Castaldo, Rossella Tomaiuolo

**Affiliations:** 1 CEINGE-Biotecnologie avanzate, Naples, Italy; 2 Dipartimento di Medicina Molecolare e Biotecnologie Mediche, Università di Napoli Federico II, Naples, Italy; 3 Laboratorio di Genetica Medica, Fondazione IRCCS Cà Granda - Ospedale Maggiore Policlnico, Mangiagalli e Regina Elena, Milan, Italy; 4 Dipartimento di Bioscienze e Territorio, Università del Molise, Isernia, Italy; Johns Hopkins School of Medicine, United States of America

## Abstract

Cystic fibrosis (CF) is the most frequent lethal genetic disorder among Caucasians. It depends on alterations of a chloride channel expressed by most epithelial cells and encoded by *CFTR* gene. Also using scanning techniques to analyze the whole coding regions of *CFTR* gene, mutations are not identified in up to 10% of CF alleles, and such figure increases in *CFTR*-related disorders (*CFTR*-RD). Other gene regions may be the site of causing-disease mutations. We searched for genetic variants in the 1500 bp of *CFTR* 3′ untranslated region, typical target of microRNA (miRNA) posttranscriptional gene regulation, in either CF patients with the F508del homozygous genotype and different clinical expression (n = 20), CF (n = 32) and *CFTR*-RD (n = 43) patients with one or none mutation after *CFTR* scanning and in controls (n = 50). We identified three SNPs, one of which, the c.*1043A>C, was located in a region predicted to bind miR-433 and miR-509-3p. Such mutation was peculiar of a *CFTR*-RD patient that had Congenital Bilateral Absence of Vas Deferens (CBAVD), diffuse bronchiectasis, a borderline sweat chloride test and the heterozygous severe F508del mutation on the other allele. The expression analysis demonstrated that the c.*1043A>C increases the affinity for miR-509-3p and slightly decreases that for the miR-433. Both miRNAs cause *in vitro* a reduced expression of CFTR protein. Thus, the c.*1043A>C may act as a mild *CFTR* mutation enhancing the affinity for inhibitory miRNAs as a novel pathogenetic mechanism in CF.

## Introduction

Cystic fibrosis (CF) is the most frequent lethal genetic disorder among Caucasians with one child in every 3000 newborns affected by disease. It depends on alterations of a chloride channel expressed by most epithelial cells and encoded by cystic fibrosis transmembrane regulator (CFTR) gene [1]. The diagnosis of CF is based on symptoms, sweat chloride and on the identification of *CFTR* mutations [2]. However, also using scanning techniques to analyze the whole coding regions of *CFTR* [3] and large gene rearrangements [4] in up to 10% of CF alleles any mutations are not identified. Such figure increases in *CFTR*-related disorders (*CFTR*-RD, [5]. It is possible that such alleles bear mutations in regulatory sequences, like those at the 3′ untranslated regions (UTR) that encode RNA sequences that bind specific microRNA (miRNA)s. These mutations may modify the affinity and thus the binding of a miRNA to the CFTR transcript, or may cause the appearance of novel target sites for miRNAs thus impairing the levels of *CFTR* expression, acting as causative mutations, or contributing to modulate the heterogeneous phenotypic expression of the disease. MicroRNAs are evolutionarily conserved, endogenous, single-stranded non-coding RNAs, 18–25 nucleotides in length [6]. These molecules regulate the expression of specific genes at post-transscriptional level [7]. The most common outcome following the formation of the miRNA/mRNA complex is the reduced expression of the gene [6], [7]. According to the miRBase, release 18 (www.mirbase.org), about 2000 miRNAs have been identified so far in humans. Recently, some miRNAs that specifically regulate *CFTR* expression have been described [8].

We searched for mutations, both in CF patients and controls, within the 3′UTR region of *CFTR* gene that could affect the interaction and therefore the regulatory activity of miRNAs, by acting as disease-causative mutations or as modifier factors of CF phenotype.

## Materials and Methods

### Ethics Approval

The study was approved by the Ethics Committee of Fondazione IRCCS Ca' Granda Ospedale Maggiore Policlinico, Milan, Italy. Written informed consent was obtained from each individual (legal guardians for minors) to use an anonymous DNA sample and clinical data for scientific studies.

### Patients and controls

We studied 95 Italian patients (Table 1) affected by CF (52 cases) or by CFTR-RD (43 cases). In detail: i) 20 CF patients homozygous for the F508del mutation, aged >18 years and classified as: severe pulmonary and liver (n = 10) or mild pulmonary and no liver (n = 10) expression as previously described [4], [9]; ii) 32 CF patients with one (28 cases) or both (4 cases) unidentified CFTR mutations after the scanning of CFTR coding regions; iii) 43 subjects with CFTR-RD (mainly CBAVD) of which 32 with one and 11 with both unidentified CFTR mutations after the whole sequencing of CFTR gene coding regions [5]. As controls, we studied 50 healthy subjects. All patients and control subjects were unrelated up to the third generation. This study was performed according to the ethical requirements of the institution, and written informed consent was obtained from each individual (legal guardians for minors) to use an anonymous DNA sample and clinical data for scientific studies.

**Table 1:** Main features of patients under study.

Diagnosis	CFTR genotype	Sweat test	Clinical expression	N of cases
CF	F508del/F508del	positive	PI, severe L, severe P	10
CF	F508del/F508del	positive	PI, no L, mild P	10
CF	Mutation/U	positive	variable	28
CF	U/U	positive	variable	4
CBAVD	Mutation/U	negative	CBAVD alone	12
CBAVD	U/U	negative	CBAVD alone	11

(PI: pancreatic insufficiency; PS: pancreatic sufficiency; L: liver; P: pulmonary)

### CFTR molecular analysis

A DNA sample was obtained from each subject (in most cases at the time of molecular analysis for diagnostic purposes). DNA was extracted from peripheral blood by standard procedures. The analysis of all CFTR coding regions (27 exons and intron-exon boundaries) was performed by gene sequencing (protocols available on request). We studied by automated gene sequencing the 3′ UTR of CFTR (starting from the 4575 last translated nucleotide up to 4575+1553). Primers and PCR conditions are available on request.

### miRNA selection

We used different computational algorithms (i.e., TargetScan, miRBase, miRanda, PITA and PicTar) to identify miRNA putative binding sites within the CFTR 3′untranslated region (3′UTR). These alghorithms are available online at: www.mirbase.org, www.targetscan.org, www.microrna.org, and www.pictar.mdc-berlin.de.

### Luciferase constructs for the 3′UTR of human *CFTR*

We cloned the 3′UTR of *CFTR* into pGL3-Control vector. To this aim, it was designed a pair of primers, both containing the site for the restriction enzyme XbaI at 5′, to amplify the entire region of the 3′UTR of *CFTR* directly from genomic DNA. Then, the PCR product was digested by XbaI and cloned into plasmid pGL3-control (Promega, USA) downstream of the luciferase cDNA (construct pLuc-*CFTR*-3′UTR). After sequencing, the construct was transfected into eukaryotic cells (HEK 293, HeLa, A549). After checking the functioning of the construct, it was co-transfected with synthetic miRNA or anti-miRNA (Qiagen, USA). Both the c.*1043A>C mutation and the overlapping miR-433 and miR-509-3p seed region mutation were performed by Site-Directed Mutagenesis using the QuikChange® II XL Site-Directed Mutagenesis Kit (Agilent Technologies) obtaining respectively the pLuc-*CFTR*-c*1043A>C and pLuc-*CFTR*-mut433-509-3p constructs.

### Western Blot analysis

Cells were lysed in 50 mM Tris, 1% Triton X-100, 150 mM NaCl and protease inhibitors (Complete tablets, Roche). Concentration of total protein extracts was determined by the BioRad method using BSA as standard. All samples, after 30 min at 37°C in Laemmli buffer, were separated by SDS-PAGE 8% gel and transferred on to Protran Nitrocellulose membrane (Whatman). The polyclonal CFTR antibody (Cell Signaling Technologies #2269) was used for CFTR protein detection and monoclonal anti-α-Tubulin (Sigma-Aldrich, T9026) for western normalization. Western blot quantification was performed using both PhotoShop and ImageJ softwares.

### MicroRNA precursor constructs and lentivirus production

In order to overexpress miR-433 and miR-509-3p in cell lines, we cloned approximately 650 bp genomic region of miR-433 gene, HGNC: 32026, and miR-509-3p gene, HGCN: 33675, in the lentiviral construct pMIRNA1, Figure S2-A (SBI System Biosciences, CA - USA). For *in vivo* expression of interfering RNA we used the pmiRZip vector (Figure S2-B), where we cloned a double-stranded oligo under the control of the constitutive RNA polymerase III H1 promoter. Once checked by sequencing, the various microRNA precursor constructs were packaged into VSV-G pseudotyped viral particles using SBI's pPACKH1 packaging plasmid Mix. Both packaging and transduction of PANC-1 cells were performed according to the manufacturer's instruction.

### Cell culture

Cell lines were purchased from ATCC (Manassas, USA). Human hepatocellular carcinoma cells (HepG2, ATCC number HB-8065), human cervix carcinoma cells (HeLa, ATCC number HB-8065), human lung carcinoma cells (A549, ATCC number CCL-185) and epithelioid carcinoma pancreatic ducts cells (PANC-1, ATCC number CRL-1469), embryonic kidney cells (HEK 293, ATCC number CRL-1573), were maintained in Dulbecco's modified Eagle's medium (Gibco Invitrogen, USA) with 10% heat-inactivated fetal bovine serum (HyClone, USA) without the addition of antibiotics; colorectal adenocarcinoma cells (Caco2, ATCC number HTB-37), were maintained in Eagle's Minimum Essential Medium with 20% heat-inactivated fetal bovine serum, normal human bronchial epithelial (NHBE) cells were purchased by Lonza (Basel, SW). cells were cultured in BEGM medium (Clonetics, Walkersville, MD), renal proximal tubular epithelial cells (RPTEC) were purchased by Lonza (Basel, SW). cells were cultured in REGM medium (Clonetics, Walkersville, MD).

### Transfection and Luciferase Assay

Transfection of HEK 293 cells with miRNA-mimics or anti-miRNA-mimics (Qiagen, Germany, EU) was performed with Attractene Transfection Reagent (Qiagen). Cells were seeded in 24-well plates and were cotransfected 24 hr after seeding with the Firefly reporter constructs described above (100 ng), the Renilla reporter plasmid pCMV-RL (50 ng), and the appropriate miRNA mimic at different concentrations (10 nM, and 30 nM). Twenty-four hours after transfection, cells were lysed and Firefly and Renilla luciferase activities were determined using the Dual-Glo Luciferase Assay System (Promega Corporation). The relative reporter activity was obtained by normalization to the Renilla luciferase activity. Each experiment was done in triplicate and at least three independent experiments were performed for each miRNA tested. Data are reported as mean (of all experiments performed)± St.Dev. Statistical significance was calculated using Student's t-test.

### MiRNA expression analysis in cells and cell lines

Total RNA was extracted from the different cells the mirVana miRNA Isolation kit (Invitrogen) according to manufacturer's protocol. The total RNA was then reverse transcribed to cDNA using the miScript Reverse Transcription kit (Qiagen). The real-time PCR quantification of mature miRNA was performed using target-specific miScript primer assay (forward primer) and the miScript SYBR Green PCR Kit, which contains the miScript Universal Primer (reverse primer) and QuantiTect SYBR Green PCR Master Mix (Qiagen). The amount of target miRNA input was normalized by human U6 snRNA.

## Results

### Sequencing analysis of the 3′UTR of CFTR and screening for functional genetic variants

**Figure 1:**
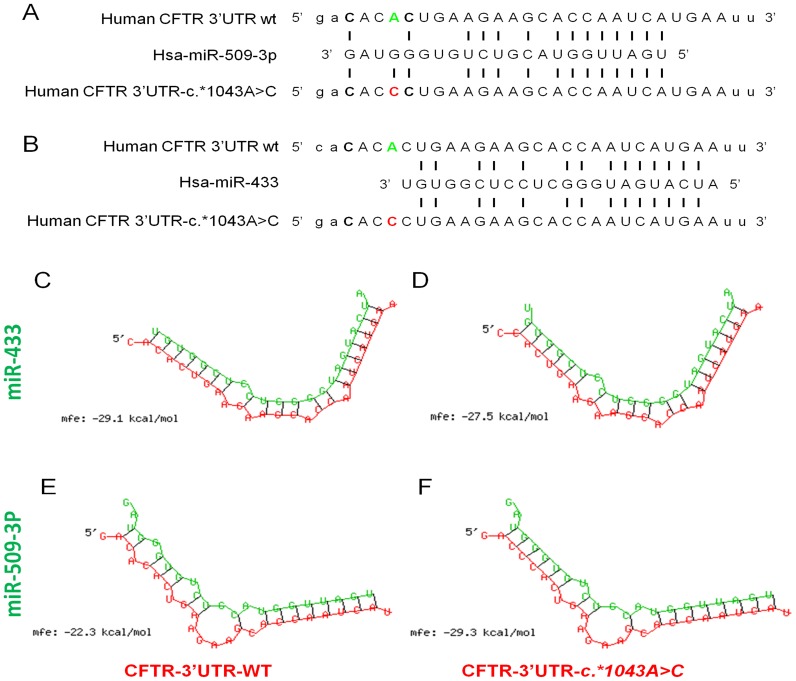
Bioinformatics prediction of c*1043A>C mutation position and effect on miRNA affinity. A–B) schematic diagram of the two *in silico* predicted miRNAs aligned with either wild-type (in green) or c.*1043A>C (in red) mutation of CFTR 3′UTR. C–D) predicted wild-type or CFTR 3′UTR-c.*1043A>C mutation Hybrid structure with miR-433. E–F) predicted wild-type or CFTR 3′UTR-c.*1043A>C mutaion Hybrid structure with miR-509-3p, for each hybrid structure is shown the mean free energy (mfe) calculated using the RNAHybrid server.

The 3′UTR (1553 bp) of the CFTR gene was sequenced from genomic DNA of 95 patients of which 52 with CF and 43 with CFTR-RD, and 50 control subjects (Table 1). We found three variants. The rs1042180 (c.*1251C>T) was found either in patients and in controls with no significant differences in the allelic frequency; the rs55831234 (c.*1283G>A) was found only in a control subject and the rs10234329 (c.*1043A>C) was identified only in a patient that had a CFTR-RD. We have focused our attention on this latter mutation present in one CFTR-RD patient, i.e., c.*1043A>C variant. First of all, because Gillen et al, have recently identified several miRNAs within the CFTR 3′UTR, we checked if any of these miRNAs fell at the site of the c*1043A>C mutation, but none of them fell on the site of our interest, so we used a variety of software available on the web to check for other miRNAs likely binding the CFTR 3′UTR close to the variant under study. The *in silico* analysis, using TargetScan5.2, predicted two putative binding sites for the miR-509-3p and miR-433. Specifically, the variant c.*1043A>C falls within the 3′ end of miR-509-3p region very close to the 3′end of miR-433 region (Figure 1, A–B). So, as the PITA algorithm [10] takes into account not only the strength of binding between miRNAs and the target-sites, but also the accessibility of the target-site, we used the PITA algorithm to analyze whether the genetic variant may change the affinity of the putative miRNAs to the target-site. The PITA algorithm predicted that the variant c.*1043A>C alters the binding of the miR-509-3p and miR-433. The same result was obtained using the RNAhybrid software (Figure 1, C–F). The two algorithms predicted about the same change in free energy between the miRNAs and the wild-type or variant form of CFTR 3′UTR. In particular, concerning the miR-433, the presence of the c.*1043A>C mutations causes a free energy lost of about 1.6 kcal/mol, while concerning the miR-509-3p, the presence of the c.*1043A>C mutation results in a free energy gain of about 7 kcal/mol (Figure 1, C–F).

### Expression of miRNA-433 and miR-509-3p in different cell lines and primary human cells

**Figure 2:**
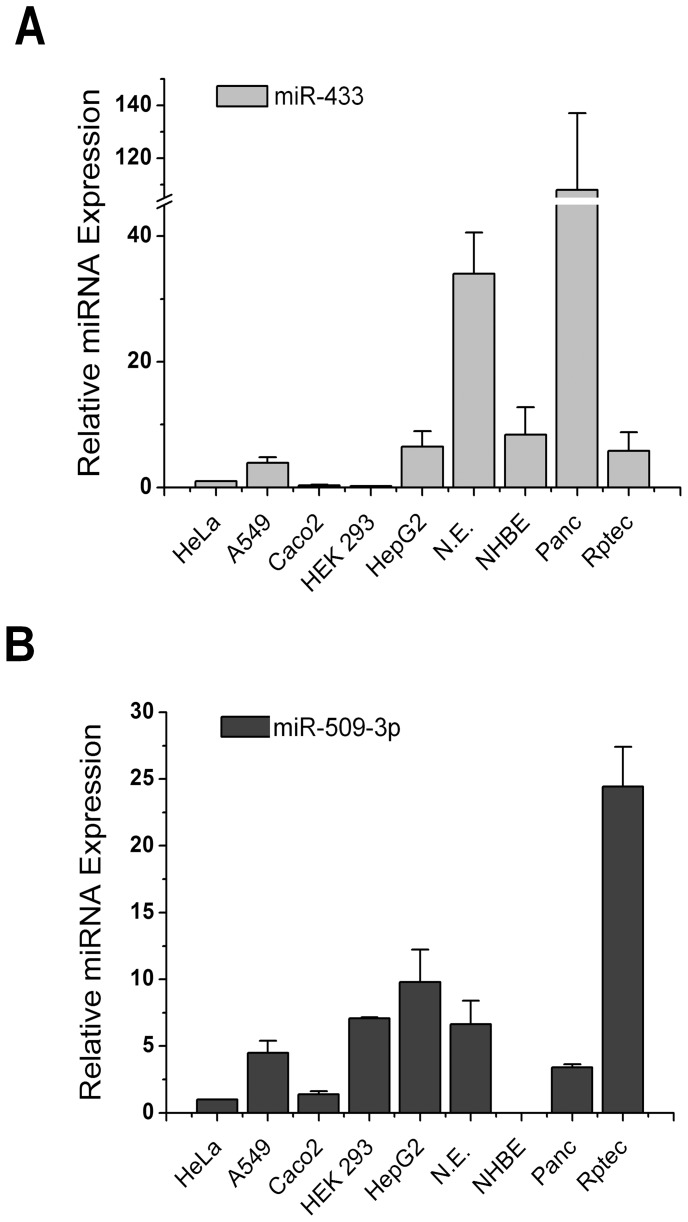
MiRNAs expression analysis. Relative expression of miR-433 A) and miR-509-3p B) in different cell line and brushed nasal epithelial cell (N.E.) compared to Hela cell line.

Before starting experiments to validate in silico predictions, we verified the expression of miR-433 and miR-509-3p in different cell lines and primary nasal epithelial cells. As shown in Figure 2, the two miRNAs are differentially expressed in various cell lines and primary cells, the RPTEC cells are those showing a high expression level of miRNA 509-3p compared to other cells, even the HepG2, HEK 293, A549 and nasal epithelial cells showed expression levels of 5–10 times higher than HeLa cells. Concerning the miRNA-433, Panc-1 cells and nasal epithelial cells showed expression levels of 40–120 times higher compared to HeLa cells. The other cell types showed levels similar to each other and approximately 5-fold compared to HeLa cells.

### Validation of miRNA Target Sites in the CFTR 3′UTR

**Figure 3:**
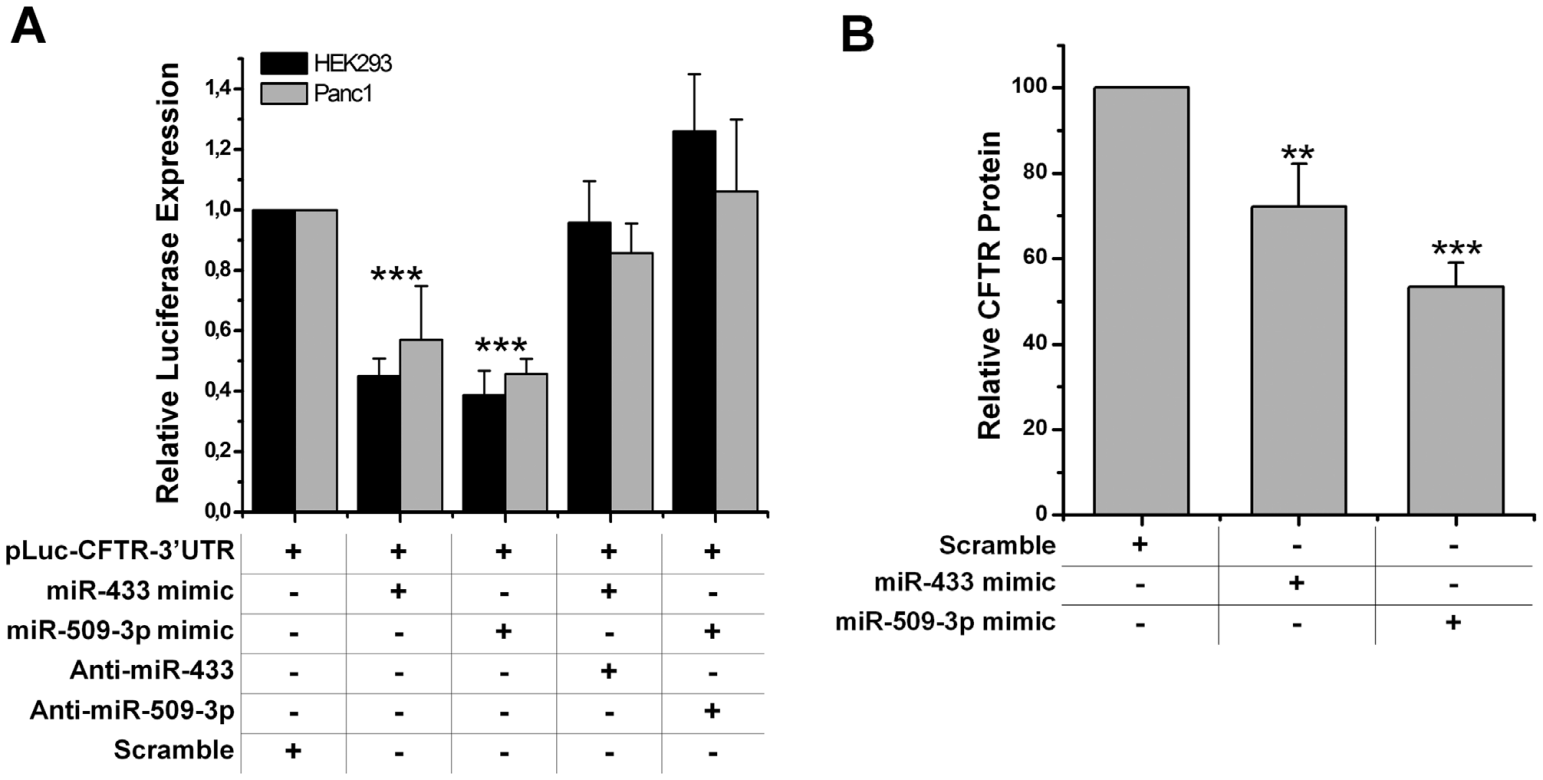
Effect of miR-433 and miR-509-3p on *CFTR*-3′UTR and CFTR protein expression.A : luciferase expression from pLuc-CFTR-3′UTR co-transfected with relative miRNA mimics and miRNA mimics plus anti-miRNA both in HEK293 and Panc1 cell lines**,** ***p<0.001 **B**: expression of CFTR protein is decreased to about 25% in Panc1 cells transfected with miR-433 mimic (** p<0.01) and about 45% in miR-509-3p (*** p<0.001) transfected cells.

**Figure 4:**
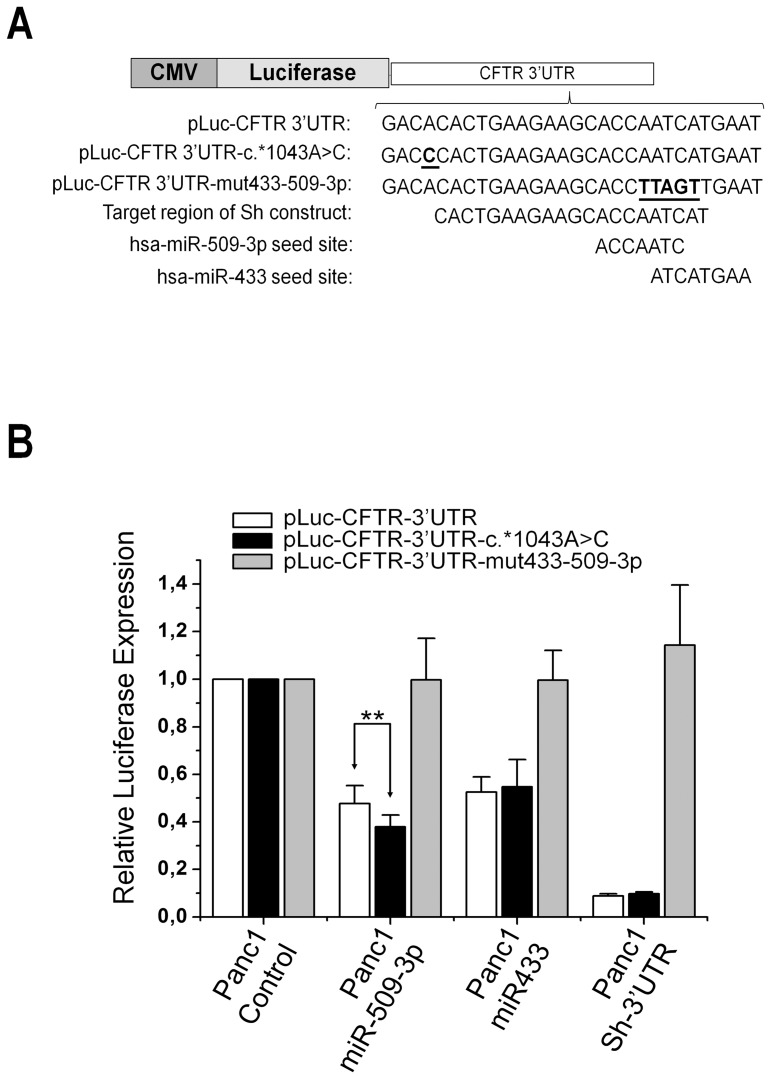
Experimental evaluation of c*1043A>C mutation effect on miRNAs affinity.A : luciferase reporter constructs derived from pGL3-Control vector. Underlined and in bold is shown the location of the variant c.*1043A>C or altered nucleotide bases to destroy the seed overlapping region of miR-433 and miR-509-3p in the CFTR 3′UTR. **B**: luciferase expression from pLuc-CFTR-3′UTR or mutated form constructs transient transfected in different Panc-1 cells stably espressing the miR-433, miR-509-3p or the sh-3′UTR. Data reported here are the means ± St.Dev., **P<0.01

Functional validation of the 2 predicted miRNA target sites containing the identified genetic variants was performed using a dual-luciferase assay in HEK 293 and in Panc1 cell lines. A luciferase-reporter construct containing the wild-type 3′UTR of CFTR was co-transfected with the miRNAs mimics of interest. As shown in Figure 3A, a statistically significant reduction of the luciferase activity was observed for both the miRNAs used (miR-509-3p, miR-433) when compared with control mimics. The down regulation observed ranged from a reduction of the luciferase expression of about 50% in the case of miR-433, up to 60% for the miR-509-3p. A western blot analysis was performed to check the levels of CFTR protein in Panc1 cells transient transfected with the two miRNAs. Both miRNAs were able, although to a different extent, to significantly reduce the endogenous levels of CFTR protein (Figure 3B). To further validate and to mimic a more physiological context, where different miRNAs may be expressed at high levels from their genomic loci, we cloned the genes of miR-433 and miR-509-3p in lentiviral vectors. We also constructed a lentiviral vector expressing a short hairpin RNA (shRNA) against the target region of both miR-433 and miR-509-3p (Figure 4A). Then, we produced lentiviral particles with which we transduced Panc 1 cells. So we obtained Panc 1 cells stably expressing each of the two miRNAs of interest or the shRNA (Panc-miR-433, Panc-miR-509-3p, Panc-sh-3′UTR). Thus, we transient transfected each of these cell lines with the wild-type construct of CFTR 3′UTR. The levels of luciferase in cells expressing high levels of miR-433 or miR-509 compared to not transduced cells were comparable to those obtained using the miRNA mimics (Figure 4B, white rectangles), the residual luciferase activity in Panc-sh-3′UTR was close to 10% compared to the control. The same results were obtained in HEK293 cell line.

### Effect of the c.*1043A>C mutation on miRNA Target Sites in the CFTR 3′UTR

To verify the RNAhybrid and PITA *in silico* predictions on the effect of c.*1043A>C mutation on the miRNAs-target duplex, we introduced by site-directed mutagenesis the (C) variant within the 3′UTR region of the construct pLuc-CFTR-3′UTR. Thus, we transient transfected the pLuc-CFTR-c.*1043A>C, bearing the (C) variant, in different Panc 1 cells stably expressing the various miRNAs or shRNA. As shown in Figure 4B the (C) variant differentially affects the miRNAs-target duplex. In particular, the luciferase activity of pLuc-CFTR-c.*1043A>C was significant reduced up to 22% (P<0.01), in Panc-509-3p compared to the construct pLuc-CFTR-3′UTR. Instead, no significant change was observed between the two constructs transfected into Panc-433 cells. Similarly, either the construct pLuc-CFTR-3′UTR and the construct pLuc-CFTR-c.*1043A>C have a residual luciferase activity of about 10% when transfected in Panc-sh-3′UTR. To further verify the specificity of action of the two miRNAs we have destroyed the two overlapping target seed regions through site-directed mutagenesis Figure 4A, (construct pLuc-CFTR-3′UTR-mut433-509-3p). The luciferase levels resulting from this construct were not affected by either miRNA or sh-3′UTR activity and was perfectly comparable to that resulting from transfected Panc 1 cells.

## Discussion

We identified a SNP in a CFTR-RD patient, already known as rs10234329 that causes an A>C change at the 4575+1043 nucleotide within the 3′ UTR of the *CFTR* gene. The *in silico* prediction indicated that such mutation, the c.*1043A>C, variant is located in the target site of two miRNAs, i.e., the miR-433 and the miR-509-3p, even if two previous studies did not consider such miRNAs as CFTR transcript modulators [8], [11]. The *in vitro* analysis demonstrated that the variant significantly enhances the affinity of the CFTR transcript for the miR-509-3p (that we demonstrated to inhibit CFTR expression). The patient had Congenital Bilateral Absence of Vas Deferens (CBAVD), diffuse bronchiectasis, pancreatic sufficiency and a borderline sweat chloride test (i.e., 41 mEq/L). He was heterozygous for the severe F508del mutation and the analysis of one parent revealed that the F508del was *in trans* with the c.*1043A>C mutation. Finally, the variant was not identified in alleles from 100 control subjects. All these data strongly suggest that the c.*1043A>C mutation may have a role in the pathogenesis of CF, impairing the regulation of gene expression, and thus acting as a mild causing disease mutation, since *CFTR-RD* patients typically are compound heterozygous for a severe and a mild CFTR mutation [5]. As regards the miR-433, the presence of the mutation c.*1043A>C, does not significantly influence its action, as expected from the *in silico* prediction. Even if this is the first report of such mechanism in cystic fibrosis (and more extensively in classic mendelian inherited diseases) similar mechanisms are becoming increasingly prevalent; in an attempt to find gene variants responsible for renal cell carcinoma (RCC), Wirsing et al. found that the rs11574744 SNP in the 3′UTR region of the HNF4A gene leads higher levels of HNF4A protein by destroying a target site for miR-34a [12]. Similarly, a SNP in a let-7 miRNA binding site in the KRAS 3′UTR increases Non-Small Cell Lung Cancer risk [13].

On the contrary, we did not identify mutations within the region at the 3′UTR of *CFTR* gene in none of 20 CF patients, homozygous for the F508del mutation, bearing a discordant pulmonary or liver expression. Of course, these data are preliminar because of the low number of patients (although carefully classified on the basis of the phenotype). Going to patients with one or both unidentified mutations, we did not identify peculiar variants within the 3′UTR CFTR region with the exception of the rs1042180, causing an C>T change at the 4575+1251 nucleotide in the 3′ UTR region, that was found in a large percentage of alleles from either CF and CFTR-RD patients and control subjects. Finally, we found the rs55831234 (c.*1283G>A) only in a control subject and in none chromosome from patients. Of course, both mutations c.*1251C>T and c.*1283G>A, even if present in control subjects, may nevertheless play a role in the expression of *CFTR* gene. This may be due not only to the action of miRNAs, but also to an effect on the accessibility to various factors involved in the conformation, translation and stability of CFTR mRNA. Of course, also for these mutations, it would be useful to understand whether they may have a pathological role.

Cystic fibrosis is characterized by a strong clinical heterogeneity. A number of factors affect this clinical variability; i.e: genotype, environmental factors, modifier genes. Our data suggest a new possible molecular mechanism in its pathogenesis. The presence of SNPs in the 3′UTR may cause a variable phenotype, not only in terms of severity but also in terms of organ specificity. In particular, at least two different mechanisms lead to this variability. First, the aberrant expression of miRNAs, whose target sequence is present in the 3′UTR of the *CFTR* gene, may decrease the expression of CFTR protein in specific organs. Secondly, as supported by our data, a decrease of CFTR protein expression can be caused by the presence of SNPs that increase the affinity for a specific miRNA in the 3′UTR of the *CFTR* gene. In addition, our data suggest that the molecular analysis of the 3′UTR region should be performed in all cases in which clinical symptoms are present but no mutation has been identified.

## Supporting Information

Figure S1 **Repression of CFTR protein expression in Panc1 cells.**Western blot analysis of CFTR protein expression in Panc1 cells using a Cell Signaling CFTR antibody after transient transfection of the indicated miRNA mimics. The molecular mass in kDa is indicated on the left-hand side.
(TIF)

Figure S2 **Plasmids used for pre-miR and Sh expression.**In panel A is shown the pMIRNA1 vector for the miR-433 and miR-509-3p expression, while in the panel B is shown the pmiRZip vector for the interfering RNA expression. Both plasmids were purchased from System Biosciences, SBI, CA–USA. In panel C are shown the oligos used for the amplification of the genomic region of both miR-433 and miR-509-3p, containing the XbaI restriction site at the 5′ end and the XhoI restriction site at the 3′ end. Further, the oligos pair for the interfering RNA is shown, with the sense region in red-bold and the antisense region in bold characters.
(TIF)
