# Supplementary figures and images for: Gene Mutation in MicroRNA Target Sites of CFTR Gene: A Novel Pathogenetic Mechanism in Cystic Fibrosis?
Source: PLoS One. 2013 Mar 26;8(3):e60448. doi: 10.1371/journal.pone.0060448 (PMC3608608; doi:10.1371/journal.pone.0060448)

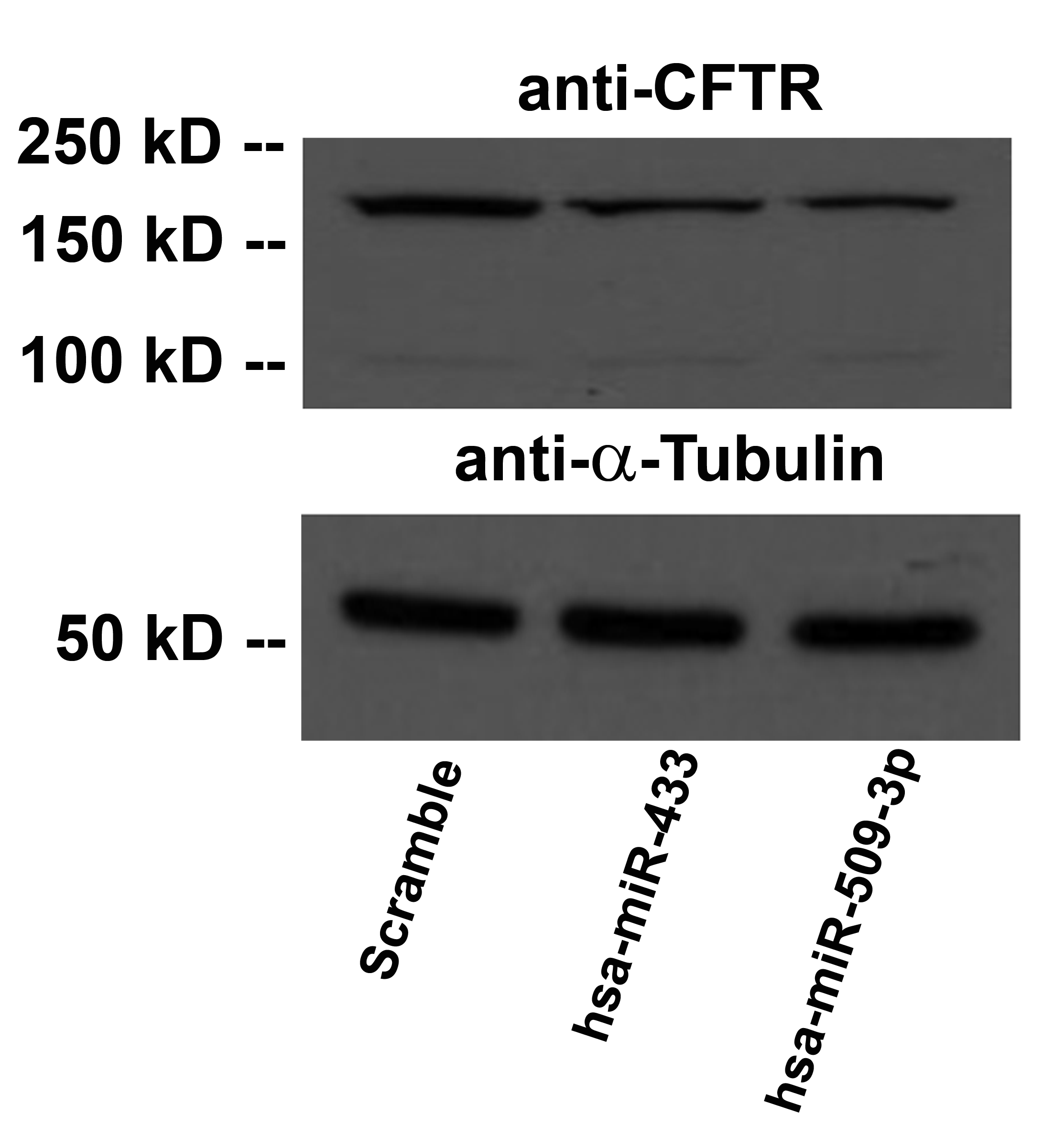

Supplement: Figure S1 — Repression of CFTR protein expression in Panc1 cells.Western blot analysis of CFTR protein expression in Panc1 cells using a Cell Signaling CFTR antibody after transient transfection of the indicated miRNA mimics. The molecular mass in kDa is indicated on the left-hand side. (TIF) [file pone.0060448.s001.tif]

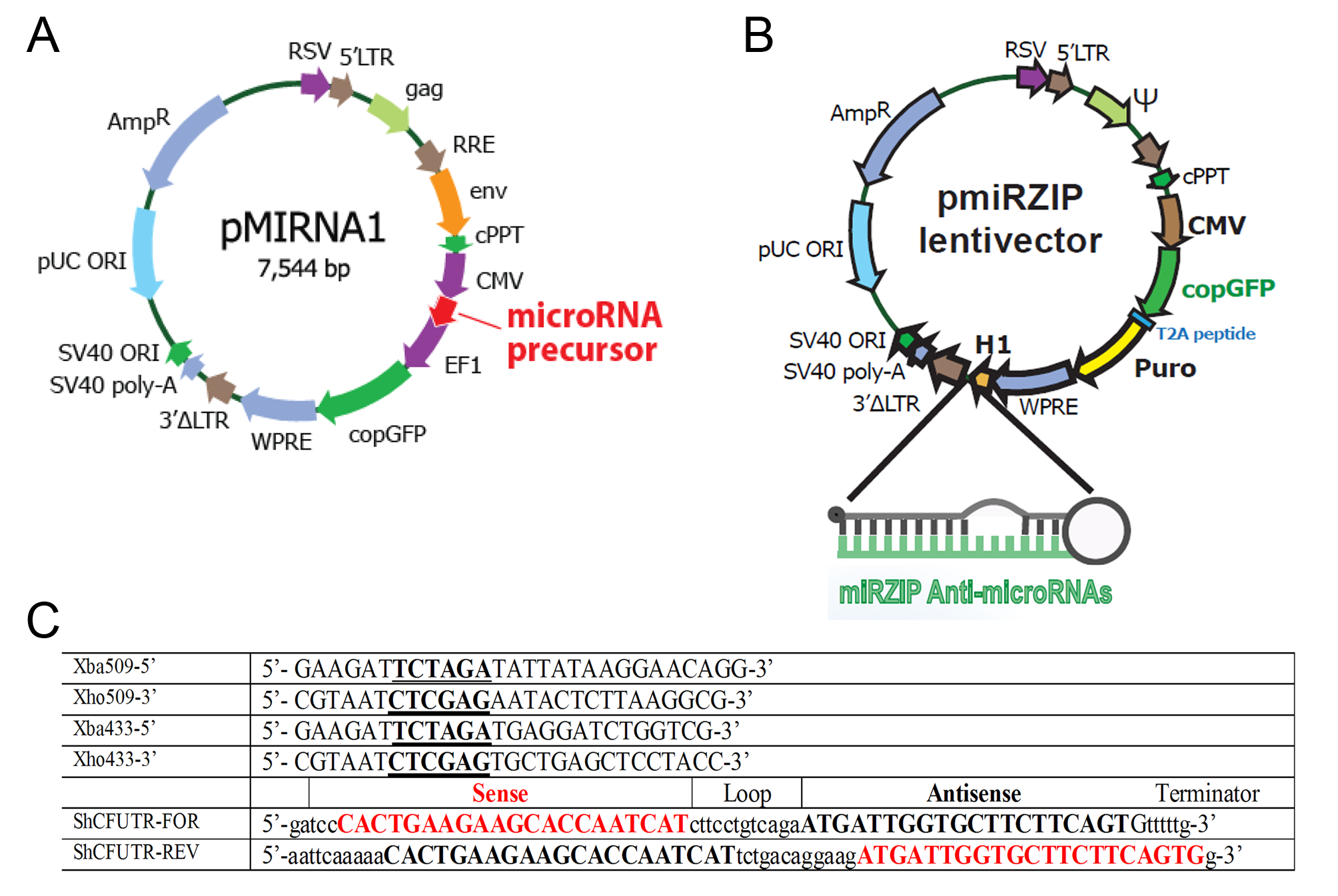

Supplement: Figure S2 — Plasmids used for pre-miR and Sh expression.In panel A is shown the pMIRNA1 vector for the miR-433 and miR-509-3p expression, while in the panel B is shown the pmiRZip vector for the interfering RNA expression. Both plasmids were purchased from System Biosciences, SBI, CA–USA. In panel C are shown the oligos used for the amplification of the genomic region of both miR-433 and miR-509-3p, containing the XbaI restriction site at the 5′ end and the XhoI restriction site at the 3′ end. Further, the oligos pair for the interfering RNA is shown, with the sense region in red-bold and the antisense region in bold characters. (TIF) [file pone.0060448.s002.tif]
